# Poly[zinc(II)-[μ-1,4-bis­(imidazol-1-yl­methyl)benzene]-μ-4,4′-oxydibenzoato]

**DOI:** 10.1107/S1600536808023982

**Published:** 2008-08-06

**Authors:** Chun-Hui Yu

**Affiliations:** a Department of Chemistry, College of Chemistry and Biology, Beihua University, Jilin City 132013, People’s Republic of China

## Abstract

In the title compound, [Zn(C_14_H_8_O_5_)(C_14_H_14_N_4_)]_*n*_, the coordination polyhedron around each Zn^II^ atom is a distorted tetra­hedron. The ligands bridge the Zn atoms to form a two-dimensional (4,4)-network.

## Related literature

For related literature, see Batten & Robson (1998 ▶); Ma *et al.* (2003 ▶).
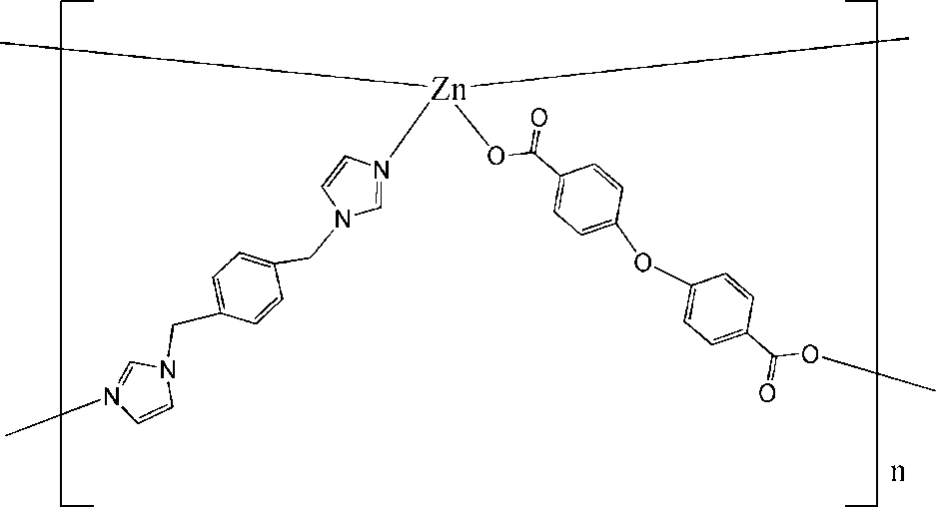


## Experimental

### 

#### Crystal data

[Zn(C_14_H_8_O_5_)(C_14_H_14_N_4_)]
*M* *_r_* = 559.87
Monoclinic, 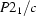

*a* = 6.1608 (9) Å
*b* = 25.811 (4) Å
*c* = 16.185 (3) Å
β = 92.503 (2)°
*V* = 2571.2 (7) Å^3^
*Z* = 4
Mo *K*α radiation
μ = 1.00 mm^−1^
*T* = 293 (2) K
0.33 × 0.25 × 0.19 mm

#### Data collection

Bruker APEX CCD area-detector diffractometer
Absorption correction: multi-scan (*SADABS*; Bruker, 1998 ▶) *T*_min_ = 0.718, *T*_max_ = 0.826
14194 measured reflections
5057 independent reflections
3906 reflections with *I* > 2σ(*I*)
*R*_int_ = 0.047

#### Refinement

*R*[*F* ^2^ > 2σ(*F* ^2^)] = 0.061
*wR*(*F* ^2^) = 0.159
*S* = 1.07
5057 reflections
343 parameters
H-atom parameters constrained
Δρ_max_ = 1.43 e Å^−3^
Δρ_min_ = −0.31 e Å^−3^

### 

Data collection: *SMART* (Bruker, 1998 ▶); cell refinement: *SAINT* (Bruker, 1998 ▶); data reduction: *SAINT* program(s) used to solve structure: *SHELXS97* (Sheldrick, 2008 ▶); program(s) used to refine structure: *SHELXL97* (Sheldrick, 2008 ▶); molecular graphics: *SHELXTL* (Sheldrick, 2008 ▶); software used to prepare material for publication: *SHELXTL*.

## Supplementary Material

Crystal structure: contains datablocks global, I. DOI: 10.1107/S1600536808023982/bt2755sup1.cif

Structure factors: contains datablocks I. DOI: 10.1107/S1600536808023982/bt2755Isup2.hkl

Additional supplementary materials:  crystallographic information; 3D view; checkCIF report

## Figures and Tables

**Fig. 1.**
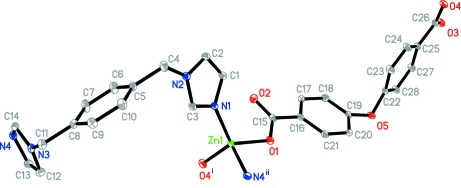
The structure of (I), showing the atomic numbering scheme. Displacement ellipsoids are drawn at the 30% probability level. Symmetry codes: (i) -1 - x, 1/2 + y, 1/2 - z; (ii) x, 1/2 - y, 1/2 + z.

**Fig. 2.**
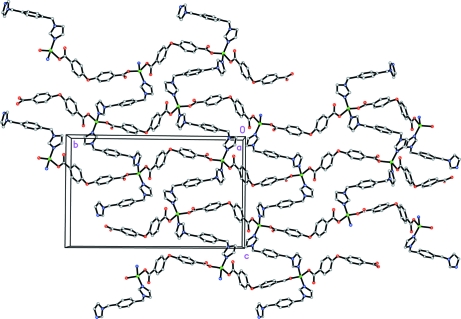
View of the chain structure of (I).

## supplementary crystallographic information

### Comment

Metal–organic frameworks is currently of great interest because of their interesting structures and potential applications. As a good candidate for rigid rod-like spacers in the construction of metal–organic polymers, 4,4'-bipyridine has been relatively well known and has shown hundreds of interesting supramolecular architectures (Batten & Robson, 1998). However, the flexible ligands such as 1,4-bis(imidazol-1-ylmethyl)benzene (*L*) has not been well explored to date (Ma *et al.*, 2003). In this work, I selected 4,4'-oxybis(benzoic acid) (H_2_oba) and *L* as linkers, generating a new coordination polymer, [Zn(oba)(*L*)], (I), which is reported here.

In compound (I) each Zn^II^ atom is four-coordinated by two N atoms from one *L* ligand, and two O atoms from two oba carboxylate anions in a distorted tetrahedral coordination sphere (Fig. 1). The two neighbouring Zn^II^ atoms are bridged by the oba and *L* ligands to form a two-dimensional (4,4) network (Fig. 2).

### Experimental

A mixture of H_2_oba (0.5 mmol), *L* (0.5 mmol), NaOH (1 mmol) and ZnCl_2_.6H_2_O (0.5 mmol) was suspended in 12 ml of deionized water and sealed in a 20 ml Teflon-lined autoclave. Upon heating at 170°C for one week, the autoclave was slowly cooled to room temperature. The crystals were collected, washed with deionized water and dried.

### Refinement

H atoms were generated geometrically and refined as riding atoms with C—H = 0.93 Å and *U*_iso_(H) = 1.2 times *U*_eq_(C).

### Crystal data

**Table:**

[Zn(C_14_H_8_O_5_)(C_14_H_14_N_4_)]	*F*_000_ = 1152
*M**_r_* = 559.87	*D*_x_ = 1.446 Mg m^−^^3^
Monoclinic, *P*2_1_/*c*	Mo *K*α radiation λ = 0.71073 Å
Hall symbol: -P 2ybc	Cell parameters from 5057 reflections
*a* = 6.1608 (9) Å	θ = 1.9–26.1º
*b* = 25.811 (4) Å	µ = 1.00 mm^−^^1^
*c* = 16.185 (3) Å	*T* = 293 (2) K
β = 92.503 (2)º	Block, colourless
*V* = 2571.2 (7) Å^3^	0.33 × 0.25 × 0.19 mm
*Z* = 4	

### Data collection

**Table:**

Bruker APEX CCD area-detector diffractometer	5057 independent reflections
Radiation source: fine-focus sealed tube	3906 reflections with *I* > 2σ(*I*)
Monochromator: graphite	*R*_int_ = 0.047
*T* = 293(2) K	θ_max_ = 26.1º
φ and ω scans	θ_min_ = 1.5º
Absorption correction: multi-scan(SADABS; Bruker, 1998)	*h* = −7→6
*T*_min_ = 0.718, *T*_max_ = 0.826	*k* = −31→27
14194 measured reflections	*l* = −19→19

### Refinement

**Table:**

Refinement on *F*^2^	Secondary atom site location: difference Fourier map
Least-squares matrix: full	Hydrogen site location: inferred from neighbouring sites
*R*[*F*^2^ > 2σ(*F*^2^)] = 0.061	H-atom parameters constrained
*wR*(*F*^2^) = 0.159	*w* = 1/[σ^2^(*F*_o_^2^) + (0.0761*P*)^2^ + 2.2361*P*] where *P* = (*F*_o_^2^ + 2*F*_c_^2^)/3
*S* = 1.07	(Δ/σ)_max_ < 0.001
5057 reflections	Δρ_max_ = 1.43 e Å^−^^3^
343 parameters	Δρ_min_ = −0.31 e Å^−^^3^
Primary atom site location: structure-invariant direct methods	Extinction correction: none

### Special details

**Table:**

Geometry. All e.s.d.'s (except the e.s.d. in the dihedral angle between two l.s. planes) are estimated using the full covariance matrix. The cell e.s.d.'s are taken into account individually in the estimation of e.s.d.'s in distances, angles and torsion angles; correlations between e.s.d.'s in cell parameters are only used when they are defined by crystal symmetry. An approximate (isotropic) treatment of cell e.s.d.'s is used for estimating e.s.d.'s involving l.s. planes.
Refinement. Refinement of *F*^2^ against ALL reflections. The weighted *R*-factor *wR* and goodness of fit *S* are based on *F*^2^, conventional *R*-factors *R* are based on *F*, with *F* set to zero for negative *F*^2^. The threshold expression of *F*^2^ > σ(*F*^2^) is used only for calculating *R*-factors(gt) *etc*. and is not relevant to the choice of reflections for refinement. *R*-factors based on *F*^2^ are statistically about twice as large as those based on *F*, and *R*- factors based on ALL data will be even larger.

### Fractional atomic coordinates and isotropic or equivalent isotropic displacement parameters (Å^2^)

**Table:**

	*x*	*y*	*z*	*U*_iso_*/*U*_eq_	
C1	0.1661 (7)	0.04984 (15)	0.0495 (3)	0.0273 (9)	
H1	0.0525	0.0275	0.0603	0.033*	
C2	0.3029 (7)	0.04487 (16)	−0.0136 (3)	0.0284 (9)	
H2	0.3004	0.0190	−0.0537	0.034*	
C3	0.3919 (7)	0.11368 (15)	0.0599 (3)	0.0281 (9)	
H3	0.4643	0.1434	0.0784	0.034*	
C4	0.6352 (8)	0.09401 (17)	−0.0563 (3)	0.0384 (11)	
H4A	0.6121	0.0764	−0.1089	0.046*	
H4B	0.7623	0.0789	−0.0283	0.046*	
C5	0.6768 (7)	0.15040 (16)	−0.0719 (3)	0.0312 (10)	
C6	0.5194 (8)	0.18139 (19)	−0.1091 (3)	0.0416 (12)	
H6	0.3861	0.1668	−0.1251	0.050*	
C7	0.5531 (8)	0.23363 (19)	−0.1233 (3)	0.0440 (12)	
H7	0.4425	0.2537	−0.1476	0.053*	
C8	0.7516 (7)	0.25584 (16)	−0.1014 (3)	0.0274 (9)	
C9	0.9128 (7)	0.22467 (18)	−0.0648 (3)	0.0355 (11)	
H9	1.0479	0.2388	−0.0502	0.043*	
C10	0.8738 (8)	0.17265 (18)	−0.0500 (3)	0.0379 (11)	
H10	0.9828	0.1524	−0.0249	0.045*	
C11	0.8036 (7)	0.31222 (17)	−0.1168 (3)	0.0333 (10)	
H11A	0.8759	0.3266	−0.0675	0.040*	
H11B	0.9032	0.3145	−0.1614	0.040*	
C12	0.4828 (8)	0.36811 (18)	−0.0836 (3)	0.0393 (12)	
H12	0.5095	0.3720	−0.0269	0.047*	
C13	0.3094 (8)	0.38615 (17)	−0.1294 (3)	0.0349 (11)	
H13	0.1968	0.4057	−0.1091	0.042*	
C14	0.5102 (7)	0.34527 (16)	−0.2128 (3)	0.0272 (9)	
H14	0.5639	0.3306	−0.2603	0.033*	
C15	−0.2311 (7)	0.04825 (15)	0.2220 (3)	0.0269 (9)	
C16	−0.3735 (7)	0.01450 (15)	0.2723 (3)	0.0272 (9)	
C17	−0.5687 (7)	−0.00464 (16)	0.2373 (3)	0.0312 (10)	
H17	−0.6076	0.0032	0.1826	0.037*	
C18	−0.7053 (7)	−0.03508 (17)	0.2825 (3)	0.0332 (10)	
H18	−0.8353	−0.0475	0.2587	0.040*	
C19	−0.6446 (7)	−0.04668 (15)	0.3635 (3)	0.0301 (10)	
C20	−0.4550 (8)	−0.02835 (16)	0.4000 (3)	0.0322 (10)	
H20	−0.4174	−0.0366	0.4547	0.039*	
C21	−0.3190 (7)	0.00262 (16)	0.3547 (3)	0.0305 (10)	
H21	−0.1910	0.0155	0.3795	0.037*	
C22	−0.8501 (7)	−0.12400 (15)	0.3885 (3)	0.0291 (9)	
C23	−0.7128 (7)	−0.15793 (17)	0.3502 (3)	0.0370 (11)	
H23	−0.5768	−0.1471	0.3344	0.044*	
C24	−0.7814 (7)	−0.20848 (16)	0.3356 (3)	0.0350 (11)	
H24	−0.6888	−0.2318	0.3111	0.042*	
C25	−0.9857 (7)	−0.22472 (15)	0.3571 (3)	0.0268 (9)	
C26	−1.0669 (7)	−0.27912 (16)	0.3427 (3)	0.0294 (10)	
C27	−1.1208 (7)	−0.18936 (16)	0.3946 (3)	0.0333 (10)	
H27	−1.2587	−0.1997	0.4090	0.040*	
C28	−1.0534 (7)	−0.13930 (17)	0.4106 (3)	0.0340 (10)	
H28	−1.1446	−0.1161	0.4360	0.041*	
N1	0.2223 (6)	0.09330 (12)	0.0948 (2)	0.0258 (8)	
N2	0.4459 (6)	0.08575 (13)	−0.0061 (2)	0.0270 (8)	
N3	0.6107 (6)	0.34292 (13)	−0.1381 (2)	0.0281 (8)	
N4	0.3250 (6)	0.37110 (13)	−0.2109 (2)	0.0282 (8)	
O1	−0.0854 (5)	0.07421 (11)	0.26198 (18)	0.0326 (7)	
O2	−0.2634 (5)	0.05027 (12)	0.14567 (18)	0.0353 (7)	
O3	−1.2588 (5)	−0.28926 (12)	0.3550 (2)	0.0393 (8)	
O4	−0.9314 (5)	−0.31274 (11)	0.31674 (19)	0.0331 (7)	
O5	−0.7843 (5)	−0.07438 (11)	0.41306 (19)	0.0380 (8)	
Zn1	0.09373 (8)	0.120902 (17)	0.19746 (3)	0.02586 (17)	

### Atomic displacement parameters (Å^2^)

**Table:**

	*U*^11^	*U*^22^	*U*^33^	*U*^12^	*U*^13^	*U*^23^
C1	0.028 (2)	0.022 (2)	0.032 (2)	−0.0029 (17)	−0.0013 (18)	−0.0012 (17)
C2	0.030 (2)	0.026 (2)	0.029 (2)	0.0022 (18)	−0.0012 (18)	−0.0039 (17)
C3	0.032 (2)	0.020 (2)	0.032 (2)	0.0034 (17)	0.0039 (19)	−0.0014 (17)
C4	0.039 (3)	0.028 (2)	0.049 (3)	0.004 (2)	0.023 (2)	0.004 (2)
C5	0.036 (3)	0.029 (2)	0.030 (2)	0.0011 (19)	0.011 (2)	0.0031 (18)
C6	0.030 (3)	0.042 (3)	0.052 (3)	−0.008 (2)	−0.004 (2)	0.011 (2)
C7	0.032 (3)	0.040 (3)	0.059 (3)	−0.003 (2)	−0.005 (2)	0.021 (2)
C8	0.026 (2)	0.031 (2)	0.026 (2)	0.0028 (18)	0.0033 (17)	0.0044 (17)
C9	0.025 (2)	0.038 (3)	0.044 (3)	0.001 (2)	−0.003 (2)	0.001 (2)
C10	0.032 (3)	0.035 (3)	0.047 (3)	0.008 (2)	−0.001 (2)	0.008 (2)
C11	0.024 (2)	0.037 (3)	0.038 (3)	−0.0009 (19)	−0.0014 (19)	0.005 (2)
C12	0.052 (3)	0.035 (3)	0.031 (2)	0.001 (2)	0.003 (2)	−0.003 (2)
C13	0.040 (3)	0.029 (2)	0.036 (3)	0.003 (2)	0.010 (2)	−0.0047 (19)
C14	0.026 (2)	0.029 (2)	0.027 (2)	0.0018 (18)	0.0003 (18)	0.0035 (17)
C15	0.027 (2)	0.017 (2)	0.037 (3)	0.0043 (17)	0.0066 (19)	0.0005 (17)
C16	0.032 (2)	0.018 (2)	0.033 (2)	0.0043 (17)	0.0065 (19)	−0.0012 (17)
C17	0.040 (3)	0.026 (2)	0.028 (2)	−0.0034 (19)	0.000 (2)	0.0000 (18)
C18	0.030 (3)	0.032 (2)	0.037 (3)	−0.009 (2)	−0.001 (2)	−0.0031 (19)
C19	0.040 (3)	0.016 (2)	0.034 (2)	−0.0043 (18)	0.009 (2)	−0.0005 (17)
C20	0.048 (3)	0.024 (2)	0.025 (2)	−0.001 (2)	0.003 (2)	0.0018 (17)
C21	0.032 (2)	0.025 (2)	0.033 (2)	−0.0044 (18)	−0.0035 (19)	−0.0005 (18)
C22	0.041 (3)	0.021 (2)	0.026 (2)	−0.0033 (19)	0.0050 (19)	0.0005 (17)
C23	0.031 (3)	0.033 (2)	0.048 (3)	−0.011 (2)	0.013 (2)	0.000 (2)
C24	0.035 (3)	0.024 (2)	0.047 (3)	0.0005 (19)	0.019 (2)	−0.0029 (19)
C25	0.028 (2)	0.022 (2)	0.031 (2)	−0.0032 (17)	0.0027 (18)	0.0014 (17)
C26	0.035 (3)	0.021 (2)	0.032 (2)	−0.0009 (18)	0.0055 (19)	0.0008 (17)
C27	0.030 (2)	0.027 (2)	0.043 (3)	−0.0020 (19)	0.009 (2)	−0.0003 (19)
C28	0.036 (3)	0.025 (2)	0.042 (3)	0.0024 (19)	0.013 (2)	−0.0013 (19)
N1	0.030 (2)	0.0206 (17)	0.0268 (18)	0.0008 (15)	0.0026 (15)	−0.0004 (14)
N2	0.029 (2)	0.0230 (18)	0.0299 (18)	0.0035 (15)	0.0051 (15)	0.0005 (14)
N3	0.029 (2)	0.0268 (19)	0.0286 (19)	−0.0002 (15)	0.0015 (16)	0.0065 (15)
N4	0.030 (2)	0.0240 (18)	0.0303 (19)	0.0032 (15)	0.0033 (15)	0.0039 (15)
O1	0.0351 (18)	0.0278 (16)	0.0354 (17)	−0.0094 (13)	0.0073 (14)	0.0003 (13)
O2	0.0397 (19)	0.0369 (18)	0.0300 (17)	−0.0037 (14)	0.0068 (14)	0.0024 (13)
O3	0.0345 (19)	0.0294 (17)	0.055 (2)	−0.0072 (14)	0.0087 (16)	−0.0032 (15)
O4	0.0340 (18)	0.0234 (15)	0.0426 (18)	−0.0012 (13)	0.0088 (14)	−0.0021 (13)
O5	0.053 (2)	0.0251 (16)	0.0375 (18)	−0.0140 (14)	0.0200 (16)	−0.0062 (13)
Zn1	0.0284 (3)	0.0203 (3)	0.0292 (3)	−0.0010 (2)	0.0048 (2)	−0.00183 (19)

### Geometric parameters (Å, °)

**Table:**

C1—C2	1.357 (6)	C15—O2	1.243 (5)
C1—N1	1.376 (5)	C15—O1	1.274 (5)
C1—H1	0.9300	C15—C16	1.502 (6)
C2—N2	1.377 (5)	C16—C21	1.395 (6)
C2—H2	0.9300	C16—C17	1.397 (6)
C3—N1	1.319 (5)	C17—C18	1.383 (6)
C3—N2	1.342 (5)	C17—H17	0.9300
C3—H3	0.9300	C18—C19	1.381 (6)
C4—N2	1.466 (5)	C18—H18	0.9300
C4—C5	1.501 (6)	C19—C20	1.370 (6)
C4—H4A	0.9700	C19—O5	1.398 (5)
C4—H4B	0.9700	C20—C21	1.389 (6)
C5—C6	1.375 (6)	C20—H20	0.9300
C5—C10	1.375 (6)	C21—H21	0.9300
C6—C7	1.385 (6)	C22—C28	1.375 (6)
C6—H6	0.9300	C22—C23	1.383 (6)
C7—C8	1.383 (6)	C22—O5	1.396 (5)
C7—H7	0.9300	C23—C24	1.389 (6)
C8—C9	1.390 (6)	C23—H23	0.9300
C8—C11	1.513 (6)	C24—C25	1.385 (6)
C9—C10	1.387 (6)	C24—H24	0.9300
C9—H9	0.9300	C25—C27	1.392 (6)
C10—H10	0.9300	C25—C26	1.505 (6)
C11—N3	1.457 (5)	C26—O3	1.236 (5)
C11—H11A	0.9700	C26—O4	1.288 (5)
C11—H11B	0.9700	C27—C28	1.378 (6)
C12—C13	1.356 (7)	C27—H27	0.9300
C12—N3	1.372 (6)	C28—H28	0.9300
C12—H12	0.9300	N1—Zn1	2.002 (3)
C13—N4	1.383 (5)	N4—Zn1^i^	2.021 (4)
C13—H13	0.9300	O1—Zn1	1.965 (3)
C14—N4	1.323 (5)	O4—Zn1^ii^	1.992 (3)
C14—N3	1.336 (5)	Zn1—O4^iii^	1.992 (3)
C14—H14	0.9300	Zn1—N4^iv^	2.021 (4)
			
C2—C1—N1	109.1 (4)	C16—C17—H17	119.3
C2—C1—H1	125.4	C19—C18—C17	118.6 (4)
N1—C1—H1	125.4	C19—C18—H18	120.7
C1—C2—N2	106.1 (4)	C17—C18—H18	120.7
C1—C2—H2	126.9	C20—C19—C18	121.6 (4)
N2—C2—H2	126.9	C20—C19—O5	117.7 (4)
N1—C3—N2	110.8 (4)	C18—C19—O5	120.5 (4)
N1—C3—H3	124.6	C19—C20—C21	119.6 (4)
N2—C3—H3	124.6	C19—C20—H20	120.2
N2—C4—C5	112.3 (3)	C21—C20—H20	120.2
N2—C4—H4A	109.1	C20—C21—C16	120.3 (4)
C5—C4—H4A	109.1	C20—C21—H21	119.8
N2—C4—H4B	109.1	C16—C21—H21	119.8
C5—C4—H4B	109.1	C28—C22—C23	121.1 (4)
H4A—C4—H4B	107.9	C28—C22—O5	116.5 (4)
C6—C5—C10	117.9 (4)	C23—C22—O5	122.2 (4)
C6—C5—C4	121.0 (4)	C22—C23—C24	118.9 (4)
C10—C5—C4	121.1 (4)	C22—C23—H23	120.5
C5—C6—C7	122.1 (4)	C24—C23—H23	120.5
C5—C6—H6	119.0	C25—C24—C23	121.0 (4)
C7—C6—H6	119.0	C25—C24—H24	119.5
C8—C7—C6	119.9 (4)	C23—C24—H24	119.5
C8—C7—H7	120.1	C24—C25—C27	118.6 (4)
C6—C7—H7	120.1	C24—C25—C26	122.9 (4)
C7—C8—C9	118.5 (4)	C27—C25—C26	118.5 (4)
C7—C8—C11	123.2 (4)	O3—C26—O4	123.3 (4)
C9—C8—C11	118.3 (4)	O3—C26—C25	119.1 (4)
C10—C9—C8	120.5 (4)	O4—C26—C25	117.6 (4)
C10—C9—H9	119.7	C28—C27—C25	121.0 (4)
C8—C9—H9	119.7	C28—C27—H27	119.5
C5—C10—C9	121.2 (4)	C25—C27—H27	119.5
C5—C10—H10	119.4	C22—C28—C27	119.4 (4)
C9—C10—H10	119.4	C22—C28—H28	120.3
N3—C11—C8	112.7 (4)	C27—C28—H28	120.3
N3—C11—H11A	109.1	C3—N1—C1	106.4 (3)
C8—C11—H11A	109.1	C3—N1—Zn1	124.3 (3)
N3—C11—H11B	109.1	C1—N1—Zn1	129.2 (3)
C8—C11—H11B	109.1	C3—N2—C2	107.5 (3)
H11A—C11—H11B	107.8	C3—N2—C4	126.4 (4)
C13—C12—N3	105.8 (4)	C2—N2—C4	125.8 (4)
C13—C12—H12	127.1	C14—N3—C12	107.7 (4)
N3—C12—H12	127.1	C14—N3—C11	125.4 (4)
C12—C13—N4	109.9 (4)	C12—N3—C11	126.2 (4)
C12—C13—H13	125.1	C14—N4—C13	105.0 (4)
N4—C13—H13	125.1	C14—N4—Zn1^i^	128.0 (3)
N4—C14—N3	111.6 (4)	C13—N4—Zn1^i^	126.5 (3)
N4—C14—H14	124.2	C15—O1—Zn1	116.9 (3)
N3—C14—H14	124.2	C26—O4—Zn1^ii^	106.7 (3)
O2—C15—O1	124.1 (4)	C22—O5—C19	119.0 (3)
O2—C15—C16	119.5 (4)	O1—Zn1—O4^iii^	107.32 (12)
O1—C15—C16	116.4 (4)	O1—Zn1—N1	118.32 (13)
C21—C16—C17	118.5 (4)	O4^iii^—Zn1—N1	115.16 (13)
C21—C16—C15	121.6 (4)	O1—Zn1—N4^iv^	93.85 (13)
C17—C16—C15	119.9 (4)	O4^iii^—Zn1—N4^iv^	109.34 (13)
C18—C17—C16	121.3 (4)	N1—Zn1—N4^iv^	110.68 (14)
C18—C17—H17	119.3		

Symmetry codes: (i) *x*, −*y*+1/2, *z*−1/2; (ii) −*x*−1, *y*−1/2, −*z*+1/2; (iii) −*x*−1, *y*+1/2, −*z*+1/2; (iv) *x*, −*y*+1/2, *z*+1/2.
